# Reconstruction of fluorophore absorption and fluorescence lifetime using early photon mesoscopic fluorescence molecular tomography: a phantom study

**DOI:** 10.1117/1.JBO.27.12.126001

**Published:** 2022-12-12

**Authors:** Alexander B. Konovalov, Vitaly V. Vlasov, Sergei I. Samarin, Ilya D. Soloviev, Alexander P. Savitsky, Valery V. Tuchin

**Affiliations:** a Federal State Unitary Enterprise “Russian Federal Nuclear Center – Zababakhin All-Russia Research Institute of Technical Physics,” Snezhinsk, Russia; b Bach Institute of Biochemistry, Research Center of Biotechnology of the Russian Academy of Sciences, Moscow, Russia; c Saratov State University, Saratov, Russia

**Keywords:** fluorescence molecular tomography, fluorescence lifetime, early arriving photons, Monte Carlo method, phantom with fluorophore, reconstruction algorithm

## Abstract

**Significance:**

Fluorescence molecular lifetime tomography (FMLT) plays an increasingly important role in experimental oncology. The article presents and experimentally verifies an original method of mesoscopic time domain FMLT, based on an asymptotic approximation to the fluorescence source function, which is valid for early arriving photons.

**Aim:**

The aim was to justify the efficiency of the method by experimental scanning and reconstruction of a phantom with a fluorophore. The experimental facility included the TCSPC system, the pulsed supercontinuum Fianium laser, and a three-channel fiber probe. Phantom scanning was done in mesoscopic regime for three-dimensional (3D) reflectance geometry.

**Approach:**

The sensitivity functions were simulated with a Monte Carlo method. A compressed-sensing-like reconstruction algorithm was used to solve the inverse problem for the fluorescence parameter distribution function, which included the fluorophore absorption coefficient and fluorescence lifetime distributions. The distributions were separated directly in the time domain with the QR-factorization least square method.

**Results:**

3D tomograms of fluorescence parameters were obtained and analyzed using two strategies for the formation of measurement data arrays and sensitivity matrices. An algorithm is developed for the flexible choice of optimal strategy in view of attaining better reconstruction quality. Variants on how to improve the method are proposed, specifically, through stepped extraction and further use of *a posteriori* information about the object.

**Conclusions:**

Even if measurement data are limited, the proposed method is capable of giving adequate reconstructions but their quality depends on available *a priori* (or *a posteriori*) information. Further research aims to improve the method by implementing the variants proposed.

## 1 Introduction

Fluorescence molecular tomography (FMT)^1^ is now widely used for the imaging of small animals with implanted tumors,^2^^–^^7^ aimed at solving the problems of experimental oncology. FMT reconstructs and maps the three-dimensional (3D) spatial distributions of informative parameters such as the fluorophore concentration and the absorption coefficient, and the fluorescence yield and lifetime. Among all these parameters, fluorescence lifetime is most sensitive to changes in the molecular surrounding of fluorescent biosensors.^8^ This parameter allows us to get the most important information on the spatial-temporal characteristics of processes that occur in the cells and molecules of small animal tissues. In this sense, further development of the existing methods of fluorescence molecular lifetime tomography (FMLT)^7^^,^^9^^–^^23^ is of particular importance in order to make them more effective. Fluorescence lifetime imaging requires time-resolved measurements, which are known to be done either in time or in frequency domain. Both fluorescence lifetime imaging microscopy^8^^,^^24^^–^^27^ and FMT prefer the time-domain measurement techniques to the frequency-domain ones because in the frequency domain, modulation of light sources is technologically limited by frequencies about 2 GHz.^28^ This makes it nearly impossible to measure the entire Fourier spectrum and hence collect the frequency domain data that are equivalent to time domain data in terms of information content. However, if we talk about FMLT, it is often the time-domain approach that becomes a serious obstacle to the high-quality reconstruction of the spatial distribution of the fluorescence lifetime. This is due to a complicated dependence of the fluorescence source function on the lifetime (see, e.g., Ref. 1), which makes it extremely difficult to derive an easy-to-implement linear model of reconstruction. There are several ways to work around the problem. The first is to take measurements in the frequency domain,^9^ where the expression for the fluorescence source function can be done much simpler. In this case, one can find a function (let us call it the fluorescence parameter distribution function, FPDF), which is written in a relatively simple form and includes the sought distributions of fluorescence parameters. FPDF reconstruction for different frequency components gives a system of equations, which can be resolved for the sought distributions. The second way assumes data registration within the time domain and then changing to the frequency domain^13^ or the Laplace transform domain^10^^,^^14^^,^^16^ to facilitate the separation of fluorescence parameters. The third is based on the so-called multiplexing method,^7^^,^^11^^,^^12^^,^^15^^,^^17^^,^^18^^,^^23^ which involves reconstruction of the fluorophore absorption coefficient (or fluorophore concentration) in the time domain for different lifetime components after which the aggregate data are processed to recover information on the spatial lifetime distribution. Luo and co-workers^19^^–^^22^ have recently implemented a “direct” FMLT method based on a nonlinear reconstruction model and the solution of nonlinear equations. However, this approach requires much more computing resources. In addition, since the quality of their first lifetime reconstructions^19^ left much to be desired, the authors had to organize a multistep reconstruction procedure,^21^^,^^22^ where additional *a posteriori* information was recovered each previous step and then used on the next step.

In this paper, we propose an alternative time domain method for FMLT and explore it against phantom measurements. It appears that if use only the early arriving diffuse photons, i.e., the photons corresponding to the leading edge of the fluorescence temporal point spread function (FTPSF), then, under certain assumptions, we can simplify the expression for the fluorescence source function in the time domain. In this case, we can derive a linear reconstruction model though not for lifetime but for a relatively simple FPDF, which includes the absorption coefficient and lifetime distributions. They can then be separated by deriving and solving a system of linear algebraic equations (SLAE). It should be noted that we have recently tried to test a similar approach numerically.^29^ But we considered only the “long” source–receiver links (SR-links) (8 and 10 mm) typical for macroscopic FMT and deliberately ignored the “short” links inherent in the mesoscopic data recording mode. Sensitivity functions were determined analytically^30^ from a diffusion approximation of the radiative transfer equation. To separate fluorescence parameters, we derived a definite SLAE. The system appeared to be very sensitive to errors in the right sides, i.e., FPDF reconstruction errors, and the quality of the images we obtained was poor. Here, we present a radical modification of the approach taken in Ref. 29. We use only the SR-links inherent in the mesoscopic mode (3.3, 2.2, and 1.1 mm). Sensitivity functions for FPDF reconstructions are simulated by the Monte Carlo (MC) method. The main idea is to separate the distributions of the absorption coefficient of a fluorophore and the fluorescence lifetime by deriving an overdetermined SLAE and solving it in terms of least squares.

The paper is organized as follows. Section 2 presents the theoretical concepts of the approach we use for the separate reconstruction of the distributions of fluorescence parameters in the time domain. Section 2.1 describes the reconstruction model for FPDF. A MC approach for modeling the sensitivity functions responsible for FPDF reconstruction is presented in Sec. 2.2. In Sec. 2.3, the inverse tomography problem is formulated and solved with respect to FPDFs. Section 2.4 describes a method for separating fluorescence parameters. Section 3 presents the results of an experiment on the reconstruction of a phantom with a fluorophore. Section 3.1 gives a description of time domain measurements. The procedure for the preliminary processing of initial measurement data is presented in Sec. 3.2. Section 3.3 describes two alternative strategies used to generate measurement datasets and sensitivity matrices for FPDF reconstruction. Section 3.4 presents the results of both FPDF reconstruction and separation of fluorescence parameters. They are discussed in Sec. 4. Section 5 summarizes the results and formulates directions for further research.

## 2 Theoretical Concepts

### 2.1 Reconstruction Model for the FPDF

The time-resolved fluorescence signal exited by an instantaneous source, which emits an infinitely short pulse from a point $r_{s}$ at time $t_{s}=0$, and detected on the medium boundary at a point $r_{d}$ at time t can be written as^19^^,^^21^

$$\Gamma^{f}(r_{s},r_{d},t)∼\int_{V}\varphi^{e}(r-r_{s},t)⊗E(r,t)⊗G^{f}(r_{d}-r,t)d^{3}r,$$ (1)

where $\varphi^{e}(r,t)$ is the density of fluorescence excitation photons, E(r,t) is the fluorophore distribution term, $G^{f}(r-r^{\prime },t-t^{\prime })$ is the fluorescence Green’s function, and ⊗ is the temporal convolution operator. The convolution $\varphi^{e}(r,t)⊗E(r,t)$ is nothing more than the fluorescence source function

$$S^{f}(r,t)=\varphi^{e}(r,t)⊗E(r,t)=\frac{\gamma \mu_{\mathrm{af}}(r)}{\tau (r)}\int_{0}^{t}\varphi^{e}(r,t^{\prime })\exp(-\frac{t-t^{\prime }}{\tau (r)})dt^{\prime },$$ (2)

where γ is the fluorescence quantum yield (We assume it to be independent of spatial coordinates.), $\mu_{\mathrm{af}}(r)$ and τ(r) are the spatial distributions of the fluorophore absorption coefficient and lifetime, respectively. Our approach is based on the following asymptotic approximation to fluorescence source function Eq. (2), applicable for FTPSF leading-edge photons

$$S^{f}(r,t)\approx \frac{\gamma \mu_{\mathrm{af}}(r)\cdot 4{\mathrm{Dct}}^{2}}{{\left|r\right|}^{2}\tau (r)+4{\mathrm{Dct}}^{2}}\varphi^{e}(r,t),$$ (3)

where D and c are, respectively, the photon diffusion coefficient and the light velocity in the medium at the excitation wavelength. For the first time, approximation Eq. (3) was considered by Lyubimov^31^ but it has not been used in practice until recently. In our recent paper,^30^ we used approximation Eq. (3) to derive and numerically test a fluorophore absorption coefficient reconstruction model for macroscopic early-photon FMT. The paper gives a detailed description of how Eq. (3) is derived. Here we omit its derivation and only state the applicability conditions for approximation Eq. (3). These are two. First, the time dependence $\varphi^{e}(r,t)$ is primarily defined by the exponential factor $\exp(-{\left|r\right|}^{2}/4\mathrm{Dct})$. This is true for the photons of the FTPSF leading edge, or rather the initial section of the FTPSF before it reaches its maximum. Second, we neglect the contribution of fluorophore absorption to the attenuation of $\varphi^{e}(r,t)$. This is true if the fluorophore is distributed locally or its absorption coefficient is small compared to the medium absorption coefficient.

With approximation Eq. (3), instead of Eq. (1) we obtain for the fluorescence signal

$$\Gamma^{f}(r_{s},r_{d},t)∼\int_{V}\int_{0}^{t}\frac{4\mathrm{Dc}\gamma \mu_{\mathrm{af}}(r)}{(\frac{{\left|r\right|}^{2}}{t^{\prime 2}})\tau (r)+4\mathrm{Dc}}\varphi^{e}(r-r_{s},t^{\prime })G^{f}(r_{d}-r,t-t^{\prime })dt^{\prime }\;d^{3}r.$$ (4)

Here, ${\left|r\right|}^{2}/t^{\prime 2}$ is the squared average velocity of photon migration from $r_{s}$ to $r_{d}$, or rather the mass center velocity of the instantaneous photon distribution along their average trajectory.^32^^–^^35^ As shown in Ref. 34, this velocity remains constant for most of the scattering object. That is why the ratio $|r{|}^{2}/t^{\prime 2}$ can be replaced by ${\left|r_{d}-r_{s}\right|}^{2}/t^{2}=v^{2}(t)$ and removed from the inner integral of Eq. (4):

$$\Gamma^{f}(r_{s},r_{d},t)∼\int_{V}\frac{4{\mathrm{Dc}\gamma \mu }_{\mathrm{af}}(r)}{\tau (r)v^{2}(t)+4\mathrm{Dc}}[\int_{0}^{t}\varphi^{e}(r-r_{s},t^{\prime })G^{f}(r_{d}-r,t-t^{\prime })dt^{\prime }]d^{3}r.$$ (5)

Expression Eq. (5) can be interpreted as an equation, which describes a linear reconstruction model for the FPDF

$$f(r)=\frac{4{\mathrm{Dc}\gamma \mu }_{\mathrm{af}}(r)}{\tau (r)v^{2}(t)+4\mathrm{Dc}}.$$ (6)

The term in the square brackets of Eq. (5) is the sensitivity function responsible for the reconstruction of f(r)

$$W_{f}(r_{s},r_{d},r,t)=\int_{0}^{t}\varphi^{e}(r-r_{s},t^{\prime })G^{f}(r_{d}-r,t-t^{\prime })dt^{\prime }.$$ (7)

Using model Eqs. (5) – (7), the inverse FMLT problem for early arriving diffuse photons can be solved in two steps: (1) reconstruct f(r) for different values of v(t) and (2) separate the distributions $\mu_{\mathrm{af}}(r)$ and τ(r) by solving the system of linear algebraic equations.

Below we show how sensitivity function Eq. (7) can be calculated with the MC method.

### 2.2 Monte-Carlo-Based Simulation of Sensitivity Functions

As mentioned in Sec. 1, in this paper we consider the mesoscopic data recording regime. Unlike macroscopic FMT, the mesoscopic one (see, e.g., Refs. 5 and 6) utilizes small SR-links (from a couple of hundred microns to a few millimeters) in order to study relatively small depths of about 0.5–5 mm. The problem is that the diffusion approximation to the radiative transfer equation, which is the preferable forward model in diffuse optical tomography and macroscopic FMT, does not suit mesoscopic FMT due to the limited volume interrogation and anisotropic light propagation.^36^^,^^37^ Moreover, the propagation of early photons (both the minimally scattered and diffuse ones) is not accurately modeled by the diffusion approximation.^38^^,^^39^ In this case, the MC method can be considered as an accurate light propagation model for solving the forward problem of mesoscopic FMT.

Thus, to simulate the FTPSFs as well as the sensitivity functions we have developed an MC code (tentatively named TurbidMC), whose algorithm is in many features similar to the MC algorithms earlier developed by other scientists (see, e.g., Refs. 40 and 41). Like the other algorithms, our one is based on modelling a large number of possible trajectories of individual photons from the site they enter the medium to the site they escape it. Photon trajectories are restored from successive simulation of elementary events: scattering, absorption, reflection (or refraction) on the boundary, free path, and fluorescence. When modelling fluorescent photon production and propagation through media, we mainly oriented on the conventional model by Welch et al.^41^ We extended it to the time domain by introducing an additional phase coordinate – time, as it is done in Ref. 42. For calculating the spatial distribution of the sensitivity function, we divide the region of interest (ROI) in the scattering object by a uniform grid of voxels (volume elements) and then determine the lengths of photon trajectory sections within the voxels.

The algorithm we use to calculate the sensitivity function in form Eq. (7) is in rather detail described in Ref. 42. Assume that we are considering totally N histories. Each begins with an excitation photon, which enters the scattering object and goes on by tracking its trajectory and the trajectories of fluorescent photons it generates. In the general case when the absorption coefficients in the medium at the excitation and fluorescence wavelengths significantly differ, the expression for sensitivity function Eq. (7) can be written as

$$W_{f}(r_{s},r_{d},r_{i},t)=\overset{N}{\underset{n=1}{\sum }}\underset{k_{n}}{\sum }w_{n,0}\;\exp[-\overset{p_{k_{n}}}{\underset{i=1}{\sum }}\mu_{a}^{e}(r_{i})l_{n}(r_{i})]\times \{1-\exp[-\mu_{a}^{e}(r_{i})L_{n}(r_{i})]\}\;\times \exp[-\overset{p_{k_{n}}+q_{k_{n}}}{\underset{i=p_{k_{n}}+1}{\sum }}\mu_{a}^{f}(r_{i})l_{k_{n}}(r_{i})]1(t_{k_{n}}<t),$$ (8)

where n and $k_{n}$ are, respectively, the index of the history and the index of the fluorescent photon in history n, $w_{n,0}$ is the initial weight of the excitation photon in history n, $\mu_{a}^{e}(r_{i})$ and $\mu_{a}^{f}(r_{i})$ are absorption coefficients in voxel $r_{i}$ at excitation and fluorescence wavelengths, respectively, $l_{n}(r_{i})$ and $l_{k_{n}}(r_{i})$ are the lengths of trajectory sections in voxel $r_{i}$ for the excitation photon in history n and fluorescent photon $k_{n}$, $L_{n}(r_{i})$ is the distance the excitation photon in history n passes in the fluorophore, $p_{k_{n}}$ is the number of voxels the excitation photon in history n crosses when migrates from point $r_{s}$ into voxel $r_{i}$ and generates fluorescent photon $k_{n}$, $q_{k_{n}}$ is the number of voxels fluorescent photon $k_{n}$ crosses when migrates from voxel $r_{i}$ to point $r_{d}$, $1(t_{k_{n}}<t)=1-\Theta (t_{k_{n}}<t)$, where $t_{k_{n}}$ is the recording time of fluorescent photon $k_{n}$, and Θ(·) is the Heaviside function.

In this work, we assume $\mu_{a}^{e}=\mu_{a}^{f}=\mu_{a}$. This assumption is standard for FMT when the therapeutic transparency window (650 to 900 nm) is meant (see, e.g., Ref. 42). Then, if neglect the fluorophore contribution to absorption and expand $1-\exp[-\mu_{a}^{e}(r_{i})L_{n}(r_{i})]$ in the Taylor series, Eq. (8) can be simplified to

$$W_{f}(r_{s},r_{d},r_{i},t)=\overset{N}{\underset{n=1}{\sum }}\underset{k_{n}}{\sum }w_{n,0}\;\exp[-\overset{p_{k_{n}}+q_{k_{n}}}{\underset{i=1}{\sum }}\mu_{a}(r_{i})l_{k_{n}}(r_{i})]\mu_{a}(r_{i})L_{n}(r_{i})1(t_{k_{n}}<t).$$ (9)

This writing implies as if the fluorescent photon migrates in the medium as an excitation photon from point $r_{s}$ to point $r_{d}$. All photons in one history travel an identical initial path to the place where the fluorophore is localized. At this stage of our study, it is Eq. (9) that was programmed and used in the TurbidMC code to calculate sensitivity functions in the fluorescence regime. The program is written in C++ of standard C++11, and uses the standard code parallelization capabilities and MCTools tools^43^ developed for the convenient description of radiation transport problems in MC codes.

**Fig. 1:**
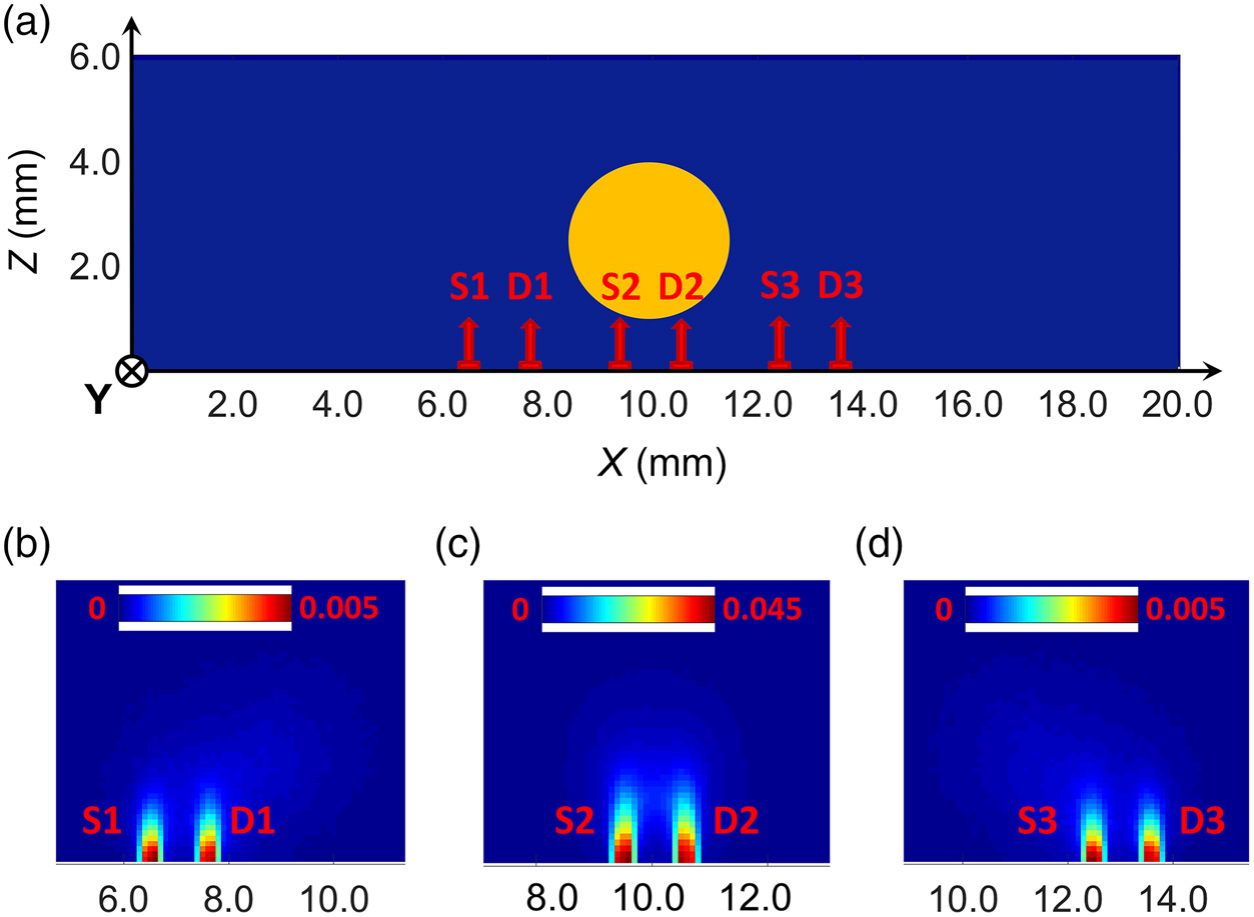
(a)–(d) Examples of sensitivity function calculations that demonstrate their dependence on the relative positions of the source–receiver pair and fluorophore. The distance between the source and receiver centers is 1.1 mm. Palette scales are graduated in relative units.

Figure 1 shows examples of sensitivity function calculations by TurbidMC for a scattering object, which simulates the phantom with the fluorophore; the experiment on its reconstruction is described in Sec. 3. In our calculations we completely simulated phantom scanning geometry with a three-channel fiber probe for one middle row of scanning (19 probe positions at a step of 0.5 mm). The values of optical and fluorescence parameters taken for calculation corresponded to the actual parameters of the phantom with fluorophore and were as follows: the absorption coefficient $\mu_{a}=0.01\;{\mathrm{mm}}^{-1}$, the scattering coefficient $\mu_{s}=2.63\;{\mathrm{mm}}^{-1}$, the refractive index n=1.521, scattering anisotropy factor g=0.62, the fluorophore absorption coefficient $\mu_{\mathrm{af}}=0.01\;{\mathrm{mm}}^{-1}$, fluorescence quantum yield γ=0.2, and fluorescence lifetime τ=900  ps. The fluorophore was shaped as a cylinder, its diameter and position corresponded to the hole in the phantom body to accommodate the fluorescent liquid [see Fig. 1(a) and Sec. 3]. Light entry and recording conditions in calculations were also matched to parameters of the three-channel fiber probe. The source and receiver were defined as 0.4-mm-diam circles oriented along the OZ axis; their centers were on the scanning line parallel to the OX axis [Fig. 1(a)]. In accordance with the three-channel fiber probe structure (see Sec. 3), the distance between the centers of the circles was set to be 3.3, 2.2, and 1.1 mm. The photon source was described by a uniform distribution of light intensity in the circle and its Gaussian distribution over an angle with a normal to the XOY plane defined for the critical angle $\vartheta_{cr}=8.2\;\deg$ that corresponded to the numerical aperture 0.2 of the fiber used in measurements. Fluorescence was collected within the same critical angle. The input pulse duration was 6 ps and the fluorescence recording time in the first series of calculations was limited by the time gate t=200  ps. About $10^{8}$ histories were tracked in each calculation. The time of calculation on the multiprocessor cluster of Russian Federal Nuclear Center – VNIITF (Shezhinsk, Russia) was about 30 to 60 min with respect to the ROI size. The maximum ROI size corresponded to the fluorescent tomogram reconstruction region, which measured $20\times 20\times 15\;{\mathrm{mm}}^{3}$ (see Sec. 2.3).

Figures 1(b)–1(d) show informative 2D sections of 3D sensitivity function distributions for three source–receiver positions [see Fig. 1(a)]: S1-D1, S2-D2, and S3-D3. Analysis of Fig. 1 shows that the results depend on the relative position of the source–receiver pair and the fluorophore. For the S2-D2 pair, the distribution is almost symmetric [Fig. 1(c)]. But if the source–receiver pair is at a distance from fluorophore, the trail distribution, which characterizes the sensitivity of deep voxels, shifts toward the fluorophore, and breaks symmetry [Figs. 1(b) and 1(d)]. It can also be seen that the amplitudes of the sensitivity functions in Fig. 1(c) and Figs. 1(b), 1(d) differ markedly. This effect suggests that the results of reconstruction depend on the conditions under which the sensitivity function is simulated in the fluorescence mode. This dependence is investigated and discussed in Sec. 4.

### 2.3 Inverse Problem Solution for the FPDF

#### 2.3.1 Setting up the inverse problem

The inverse problem with respect to the FPDF (or the problem of reconstructing the FPDF), in accordance with linear model Eqs. (5)–(7), is formulated in a standard way (see, e.g., Refs. 1 and 37) and reduces to solving the SLAE

Wf=g, (10)

where W is the sensitivity matrix that stores sensitivity functions Eq. (9) calculated for all SR-links involved in reconstruction; f is a vector, whose I elements define the sought function f(r) in the voxels ${\left\{r_{i}\right\}}_{1}^{I}$ of the 3D uniform grid; g is a vector, whose J elements represent measurement data extracted from the measured fluorescence temporal responses (FTRs, see Sec. 3). So, the dimension of W is J×I. Since we reconstructed a region of size $20\times 20\times 15\;{\mathrm{mm}}^{3}$ and the voxel was taken to be 0.1 mm in size, the grid measured 200×200×150. So, in our case, I=6000000. As for the number of the elements of the vector g (or the number of rows in the matrix W), J was determined in accordance with two strategies described in Sec. 3 and took values 361, 1083, and 722. Thus, in all cases system Eq. (10) was strongly underdetermined and its solution required reconstruction with regularization.

#### 2.3.2 Reconstruction algorithm

Now let us discuss considerations on the choice of the inversion algorithm for system Eq. (10). In the last decade, fluorescence tomography has begun to widely use the so-called compressed-sensing-like reconstruction algorithms, which minimize the $L_{p}$ norm of the sought solution, where 0≤p≤1 (see, e.g., Refs. 1, 19–22, 29, 30, and 44–56). The most popular of them are iterative shrinkage thresholding algorithms^30^^,^^45^^,^^46^^,^^50^^,^^51^^,^^53^^,^^55^^,^^56^ and algorithms with total variation (TV) regularization.^29^^,^^30^^,^^44^^,^^47^^–^^49^^,^^53^ The former are usually used to reconstruct sparse images as, e.g., in the cases where the fluorophore is localized in small vessels or a small tumor. Algorithms with TV regularization are most effective when piece-wise constant functions are reconstructed, e.g., a uniform fluorophore distribution in the tissues and organs of a small animal. In Ref. 30, we compared two compressed-sensing-like algorithms based on the standard algebraic reconstruction technique (ART),^57^ specifically ART with TV regularization (ART-TV)^58^^,^^59^ and ART with fast iterative shrinkage thresholding (ART-FIST). The latter is our modification of the known fast iterative shrinkage thresholding algorithm.^60^ It is shown in Ref. 30 that ART-FIST significantly outperforms ART-TV both in reconstruction accuracy and in convergence speed. However in Ref. 30, we tested spatial resolution and reconstructed an object with fluorescent inclusions of small sizes (0.1 to 0.5 mm), i.e., a deliberately sparse image. In the present case, the fluorophore occupies rather a large part of the phantom ROI, where it is distributed uniformly (see Sec. 3). That is why the image to be reconstructed should be qualified as piece-wise constant structured rather than sparse. In this situation, we found it appropriate to use a combination of the two algorithms investigated in Ref. 30, i.e., include TV-regularization in ART-FIST. Our new algorithm we refer to as ART-FIST-TV differs from ART-FIST in that it implements a cycle of TV iterations with the steepest descent method^58^^,^^59^ after a cycle of ART-FIST iterations. Then an external cycle is organized, where these two internal cycles work one after another. ART-FIST-TV testing shows that this hybrid algorithm converges faster and gives more stable results than ART-FIST. A step-by-step description of the ART-FIST-TV algorithm is given in Appendix.

### 2.4 Method of Fluorescence Parameter Separation

As mentioned in the Introduction, reliable separation of the distributions $\mu_{\mathrm{af}}(r)$ and τ(r) requires an overdetermined system of equations, i.e., the fluorescence parameter distribution function f(r) needs to be reconstructed for at least three values of the average photon migration velocity ${\left\{v_{m}\right\}}_{1}^{3}$. Then we will have three distributions $\{f_{m}(r){\}}_{1}^{3}$ for which, in accordance with Eq. (6), the following three equations must be satisfied:

$$\frac{4\mathrm{Dc}\gamma \mu_{\mathrm{af}}(r)}{\tau (r)v_{m}^{2}+4\mathrm{Dc}}=f_{m}(r),\;m=1,2,3.$$ (11)

Rewrite Eq. (11) as the SLAE to be solved for unknown $\mu_{\mathrm{af}}(r)$ and τ(r)

$$\left\{ \begin{array}{l} 4\mathrm{Dc}\gamma \cdot \mu_{\mathrm{af}}(r)-f_{1}(r)v_{1}^{2}\cdot \tau (r)=4{\mathrm{Dcf}}_{1}(r)\\ 4\mathrm{Dc}\gamma \cdot \mu_{\mathrm{af}}(r)-f_{2}(r)v_{2}^{2}\cdot \tau (r)=4{\mathrm{Dcf}}_{2}(r)\\ 4\mathrm{Dc}\gamma \cdot \mu_{\mathrm{af}}(r)-f_{3}(r)v_{3}^{2}\cdot \tau (r)=4{\mathrm{Dcf}}_{3}(r), \end{array}\right.$$ (12)

or

Ax=b, (13)

where $A=(\begin{array}{cc} 4\mathrm{Dc}\gamma & -f_{1}(r)v_{1}^{2}\\ 4\mathrm{Dc}\gamma & -f_{2}(r)v_{2}^{2}\\ 4\mathrm{Dc}\gamma & -f_{3}(r)v_{3}^{2} \end{array})$, $x=(\begin{array}{l} \mu_{\mathrm{af}}(r)\\ \tau (r) \end{array})$ and $b=(\begin{array}{l} 4{\mathrm{Dcf}}_{1}(r)\\ 4{\mathrm{Dcf}}_{2}(r)\\ 4{\mathrm{Dcf}}_{3}(r) \end{array})$.

It is appropriate to seek a solution for Eq. (13) in terms of least squares. Now available are a wealth of least squares algorithms (see, e.g., Ref. 61), which can successfully be used for system Eq. (13). Since the system does not exhibit singularities, which can complicate its solution (it is not underdetermined, not large, does not require the lower condition number, etc.), we can choose any with approximately equal chances of getting a true solution. We chose the iterative QR-factorization least square (LSQR) algorithm^62^ that is based on a bidiagonalization procedure by Golub and Kahan^63^ and on orthogonal QR-factorization with the modified flat rotation technique.^61^ A peculiarity of this algorithm is the use of a control parameter ω, whose value is taken to be between 0 and 1. The parameter often strongly influences calculation speed and solution accuracy, i.e., actually performs as a regularizing function. For the LSQR algorithm, the linear least square problem is stated for the extended system,

$$[\begin{array}{l} A\\ \omega I \end{array}]x=[\begin{array}{l} b\\ 0 \end{array}],$$ (14)

where I is the unit matrix, and 0 is the zero vector, and reduces to minimization of the Euclidean norm of the residual

$${\left\|[\begin{array}{l} A\\ \omega I \end{array}]x-[\begin{array}{l} b\\ 0 \end{array}]\right\|}_{2}\to \min.$$ (15)

For solving optimization problem Eq. (15), we used MATLAB, where the LSQR algorithm is implemented by the operator lsqr(·).

## 3 Phantom Reconstruction Experiment

### 3.1 Experimental Setup and Measurement Procedure

**Fig. 2:**
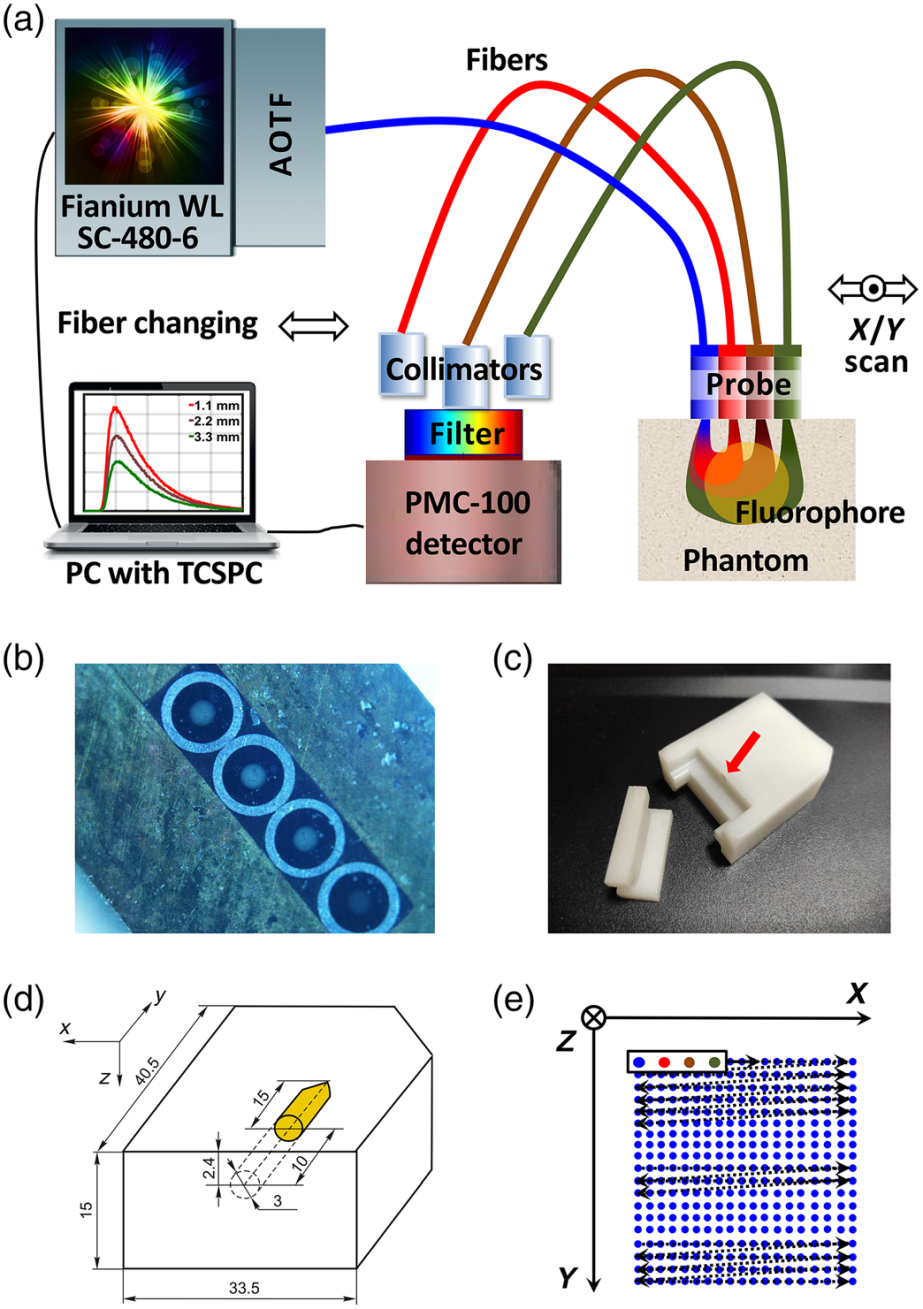
(a) Experimental setup, (b) a three-channel fiber probe, (c) the phantom taken to pieces to show the hole for fluorescent liquid, (d) the assembled phantom, and (e) phantom scanning scheme. In Fig. 2(d), all lengths are in millimeters.

The experiment for scanning a phantom with a fluorophore was done at the Bach Institute of Biochemistry, Research Center of Biotechnology of the Russian Academy of Sciences (Moscow, Russia). The experimental facility was constructed on the basis of the time correlated single photon counting (TCSPC) system (Becker & Hickl GmbH, Berlin, Germany): the PMC-100 single photon counting detector, and the SPC-150 TCSPC module. Fluorescence was excited by the pulsed supercontinuum SC-480-6 laser (Fianium UK Ltd., Southampton, UK) with the acousto-optical tunable filter (AOTF) of emitted light wavelength [Fig. 2(a)]. The pulse width was 6 ps before AOTF, the wavelength was 640 nm, and the average power on the fiber outlet was 3 mW. For fluorescence temporal response registration in reflectance geometry, we used a three-channel fiber probe with four fibers, fixed linearly with a center-to-center distance of 1.1 mm [Fig. 2(b)]. Each of the four fibers had a 400-μm-diam core and numerical aperture 0.2. The first fiber was used to inject exciting light and the other three for FTR measurement. The phantom was a parallelepiped of the homogeneous, tissue-like material INO Biomimic based on polyurethane with the addition of scattering particles of titanium dioxide [Fig. 2(c)]. Along the parallelepiped there was a cylindrical hole for the fluorescent solution [Figs. 2(c) and 2(d)]. Considering that cyanines are widely used in FMT as fluorescence agents (see, e.g., Refs. 3–5, 16, 45, and 46), Cy5 was taken to serve as a fluorophore. Its concentration was $5\cdot 10^{-7}\;\mathrm{mol}/L$. The maximal excitation and emission wavelengths of Cy5 were, respectively, 649 and 666 nm. HQ720/60 band-pass and HQ650LP blocking filters (Chroma Technology Corp., Vermont) were used for registration. The scheme of phantom scanning is shown in Fig. 2(e). The fiber probe was moved along the phantom surface with a 0.5 mm step using a micrometer translator. First, scanning was performed in the direction of the horizontal X-axis for 19 fixed positions, then the probe moved to the beginning of the next row and again ran along the X-axis, and so on along a zigzag path. A total of 19 rows were scanned on an area of $9\times 9\;{\mathrm{mm}}^{2}$. For each of the 361 probe positions, three FTR measurements were done for three SR-distances: 3.3, 2.2, and 1.1 mm. For scanning, we used three collimators (one for each of the receiving fibers) and 1 detector with the filter. The collimators were rearranged by hand for each of the 361×3=1083 excitation positions. So, the measurements were taken sequentially. In addition, in order to be able to estimate the true FTPSFs from the measured FTRs (see Sec. 3.2), the instrumental response function (IRF) was measured using the technology described, e.g., in Ref. 14.

### 3.2 Raw Measurement Data Preprocessing

According to the FPDF reconstruction model presented in Sec. 2.1, the measurement data g are the individual FTPSF counts corresponding to the chosen receiver time-gating delays (time gates) t. It is known (see, e.g., Ref. 14) that the measured FTR, ${\tilde{\Gamma }}^{f}(r_{s},r_{d},t)$, is related to FTPSF, $\Gamma^{f}(r_{s},r_{d},t)$, as

$${\overset{˜}{\Gamma }}^{f}(r_{s},r_{d},t)=C\cdot \Gamma^{f}(r_{s},r_{d},t)⊗\mathrm{IRF}(t-t_{\text{shift}})+e(r_{s},r_{d},t),$$ (16)

where C denotes the coupling factor that matches the intensity of FTR to that of FTPSF, IRF(t) is the instrumental response function, $t_{\text{shift}}$ is the stochastic shift in the time origin, and $e(r_{s},r_{d},t)$ is the noise term. So, in order to evaluate FTPSF from FTR and IRF measurements, we have to (1) neutralize noise $e(r_{s},r_{d},t)$, (2) provide deconvolution ${\tilde{\Gamma }}^{f}(r_{s},r_{d},t)$ with IRF(t), and (3) determine the proper position of the deconvolution result relative to the time origin, which we assume to correspond to the beginning of the input excitation pulse. Note that from Eq. (16), one more problem follows – the problem of finding the constant C but here we do not state and solve it. It reduces to another problem – the problem of determining the proportionality factor between the left- and right-hand sides of Eq. (5), which is solved at the stage of FPDF reconstruction through calibration calculations.

**Fig. 3:**
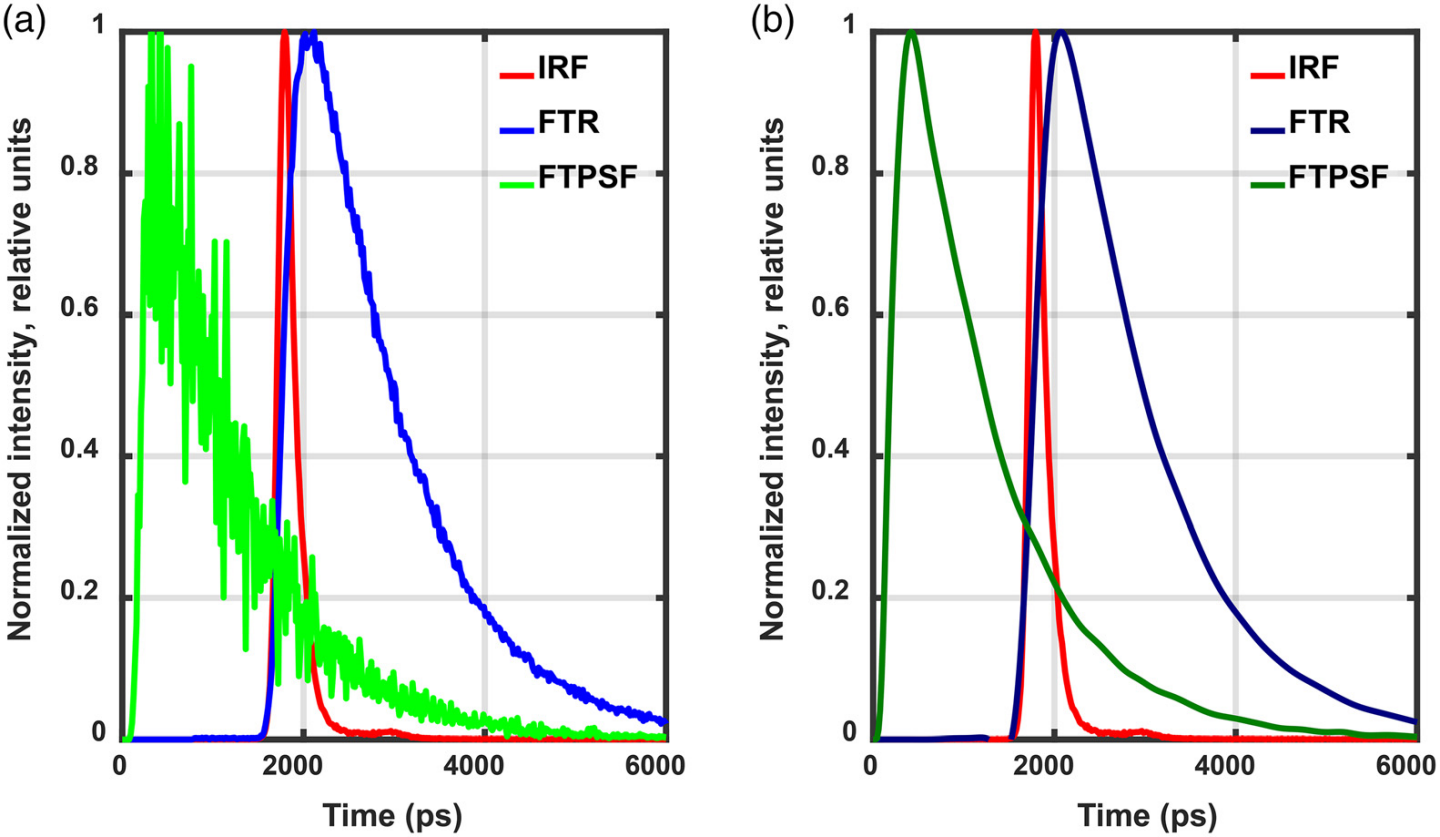
Two examples of FTR deconvolution with IRF: (a) without and (b) with noise neutralization. For ease of visual analysis, all pulse amplitudes are normalized to unity.

Unlike, e.g., Ref. 14, we work in the time domain only and use non-normalized data for reconstruction. In this situation, the problem of noise neutralization is of particular importance and cannot be ignored. This is demonstrated in Fig. 3 with two examples of deconvolution (green and bottle-green) of FTR (blue and navy-blue) with IRF (red). The first example [Fig. 3(a)] shows what is obtained if deconvolution is applied to the measured noisy FTR (blue).

It is seen that the noise present in the result of deconvolution (green) is higher in amplitude than the noise of the initial blue pulse. Such a signal holds no prospect of recovering the time-gate-based measurement datum, which will be used for reconstruction. The other example [Fig. 3(b)] demonstrates the result of deconvolution (bottle-green) of FTR after noise removal (navy-blue) with IRF. The bottle-green line is rather smooth and can in principle be used to form measurement data of any type. So, all the FTRs registered were smoothed to remove noise. Preprocessing was done with MATLAB. For noise removal, we used the Savitsky–Golay filter^64^ implemented in MATLAB by the sgolayfilt(·) operator. Deconvolution of FTRs smoothed with IRF was done using the accelerated Lucy–Richardson algorithm,^65^ which proved to perform well in processing both 1D signals and images including the fluorescent ones (see, e.g., Ref. 66). The algorithm is implemented in MATLAB by the deconvlucy(·) operator.

In order to determine the true position of the processed FTRs relative to the time origin, we performed a series of 19 calculations by the code TurbidMC to model the FTPSFs. They were modeled for the same initial data as the sensitivity functions (see Sec. 2.2). We found that the leading edge of FTPSF pulses started 30 to 40 ps after the time origin (the start of the exciting pulse) and the FTPSF maxima were within 250 to 300 ps. So, for forming the array of measurement data (see Sec. 3.3), we shifted the pulses resulted from FTR deconvolution with IRF in accordance with data from FTPSF modeling. Figure 3 shows the true (already corrected) position of deconvolution results (the green and bottle-green lines) relative to the time origin.

### 3.3 Two Strategies of Generating Measurement Data Arrays and Sensitivity Matrices for FPDF Reconstruction

So, in accordance with the fluorescence parameter separation method described in Sec. 2.4, we were able to decide how to choose three values for the average photon velocity, $\{v_{m}{\}}_{1}^{3}$, for reconstructing three FPDF distributions. To this end, it was necessary to determine the appropriate values of time gates and the strategy of forming the arrays g and W for each of the three cases. Since we only had three different values of the SR-distances R, specifically, $R_{1}=3.3\;\mathrm{mm}$, $R_{2}=2.2\;\mathrm{mm}$, and $R_{3}=1.1\;\mathrm{mm}$, the simplest strategy (hereafter strategy 1) was to take one time gate t and reconstruct the discrete FPDF distributions ${\left\{f_{m}\right\}}_{1}^{3}$ for three photon velocities $v_{1}=R_{1}/t$, $v_{2}=R_{2}/t$, and $v_{3}=R_{3}/t$, using for each reconstruction the SR-links corresponding to one SR-distance: $R_{1}$, $R_{2}$, or $R_{3}$. From our analysis of the processed FTRs (i.e., FTPSFs, see Sec. 3.2) we found that the time gate t=200  ps always corresponded to the leading edges of FTPSFs and fell within 75% to 90% of FTPSF maxima. That is why just this time, for which we did the first series of calculations to obtain the sensitivity functions (see Sec. 2.2), was taken as a single time gate for strategy 1. So, strategy 1 implied the formation of three measurement data arrays g each of length J=19×19=361, three sensitivity matrices W each of size 361×6000000, and the reconstruction of three distributions ${\left\{f_{m}\right\}}_{1}^{3}$ for the following three photon velocities ${\left\{v_{m}\right\}}_{1}^{3}$:

$$v_{1}=\frac{R_{1}}{t}=\frac{3.3}{200}=0.0165\;\mathrm{mm}/\mathrm{ps},\;v_{2}=\frac{R_{2}}{t}=\frac{2.2}{200}=0.011\;\mathrm{mm}/\mathrm{ps},\;v_{3}=\frac{R_{3}}{t}=\frac{1.1}{200}=0.0055\;\mathrm{mm}/\mathrm{ps}.$$ (17)

The sensitivity matrices W were formed using results from modeling the space-dependent sensitivity functions, which were obtained for one row of phantom scanning (see Sec. 2.2). For the other 18 rows, we repeated these sensitivity function sequences.

Unfortunately, despite its simplicity, strategy 1 has two obvious shortcomings. First, a doubt immediately rises if that limited SR-links (361) are sufficient for the reconstruction of each of the three FPDF distributions. Second, in the case where the SR-links with $R_{3}=1.1$ mm are used, sensitivity may appear insufficient for the reproduction of fluorescence parameters at depth because the depth where the fluorophore is located in the phantom is almost four times as large as $R_{3}$. In this situation, we found it appropriate to develop an alternative strategy (hereafter strategy 2), which was intended to use more SR-links for each reconstruction than in strategy 1. Set a goal to organize FPDF reconstruction for three photon velocities in such a way as to use either all SR-links, or the links corresponding to at least two SR-distances: $R_{1}$ and $R_{2}$, $R_{2}$ and $R_{3}$, or $R_{1}$ and $R_{3}$. It is important here to minimize the number of time gate values, for which it will be necessary to do resource–demanding model calculations of sensitivity functions in addition to those done for t=200  ps.

First assume that all SR-links are used for FPDF reconstruction. Assign each of the three SR-distances to its value of time gate: $R_{1}\to t_{1}$, $R_{2}\to t_{2}$, and $R_{3}\to t_{3}$ so as to satisfy the condition

$$\frac{R_{1}}{t_{1}}=\frac{R_{2}}{t_{2}}=\frac{R_{3}}{t_{3}}.$$ (18)

This is easy to do if take, e.g., $t_{1}=200\;\mathrm{ps}$

$$t_{2}=\frac{R_{2}}{R_{1}}t_{1}=\frac{2.2}{3.3}200=133\;\mathrm{ps}\;\mathrm{and}\;t_{3}=\frac{R_{3}}{R_{1}}t_{1}=\frac{1.1}{3.3}200=66\;\mathrm{ps}.$$ (19)

So, using all SR-links and the above time delays, we can reconstruct FPDF for velocity

$$v_{1}=\frac{3.3}{200}=\frac{2.2}{133}=\frac{1.1}{66}=0.0165\;\mathrm{mm}/\mathrm{ps}.$$ (20)

Now let $t_{2}=200\;\mathrm{ps}$. Then $t_{1}$, according to Eq. (18), should be >200  ps. But this is highly undesirable, as examination in Sec. 3.2 shows that this time gate is close to the maximum allowable. Therefore, we must exclude the SR-links with $R_{1}$ from this calculation. The time $t_{3}$ is calculated as

$$t_{3}=\frac{R_{3}}{R_{2}}t_{2}=\frac{1.1}{2.2}200=100\;\mathrm{ps}.$$ (21)

So, if take $t_{2}=200\;\mathrm{ps}$ and $t_{3}=100\;\mathrm{ps}$, we can reconstruct FPDF from SR-links with $R_{2}$ and $R_{3}$ for velocity

$$v_{2}=\frac{2.2}{200}=\frac{1.1}{100}=0.011\;\mathrm{mm}/\mathrm{ps}.$$ (22)

Finally, let $t_{2}=66\;\mathrm{ps}$. Then, in accordance with Eq. (18), $t_{3}$ must be smaller than 66 ps. This is also undesirable because of a high risk to appear in the region of high noise and, hence, unreliable values of FTPSFs. This means that in this calculation we must exclude the SR-links with $R_{3}$. The time $t_{1}$ is calculated as

$$t_{1}=\frac{R_{1}}{R_{2}}t_{2}=\frac{3.3}{2.2}66=100\;\mathrm{ps}.$$ (23)

That is, if we take $t_{1}=100\;\mathrm{ps}$ and $t_{2}=66\;\mathrm{ps}$, we can reconstruct FPDF from SR-links with $R_{1}$ and $R_{2}$ for velocity

$$v_{3}=\frac{3.3}{100}=\frac{2.2}{66}=0.033\;\mathrm{mm}/\mathrm{ps}.$$ (24)

So, in accordance with strategy 2, it is necessary to form one measurement data array g of length J=19×19×3=1083, two arrays g of length J=19×19×2=722, one sensitivity matrix W of size 1083×6000000, and two sensitivity matrices of size 722×6000000. In this case, it is necessary to reconstruct three FPDFs for photon velocities defined by Eqs. (20), (22), and (24). The sensitivity matrices were formed from the earlier sensitivity function calculations for t=200  ps and five series of additional calculations for the following combinations of parameters: (1) $R_{1}=3.3\;\mathrm{mm}$, $t_{1}=100\;\mathrm{ps}$; (2) $R_{2}=2.2\;\mathrm{mm}$, $t_{2}=133\;\mathrm{ps}$; (3) $R_{2}=2.2\;\mathrm{mm}$, $t_{2}=66\;\mathrm{ps}$; (4) $R_{3}=1.1\;\mathrm{mm}$, $t_{3}=100\;\mathrm{ps}$; (5) $R_{3}=1.1\;\mathrm{mm}$, $t_{3}=66\;\mathrm{ps}$. Like earlier, all these calculations were done by TurbidMC (see Sec. 2.2) for the middle row of phantom scanning. As in the case of strategy 1, we formed the matrices W by repeating these modeled sequences of sensitivity functions for the other rows of scanning. To facilitate understanding, the values of parameters characterizing strategies 1 and 2 are summarized in Table 1. In addition, the table gives the true values of FPDF calculated by Eq. (6) and expected after reconstruction at the fluorophore localization sites.

**Table 1:** Parameters of strategies 1 and 2.

Parameters	Reconstruction No.	Strategy 1	Strategy 2
Used SR-distances R (mm)	1	3.3	3.3; 2.2; 1.1
	2	2.2	2.2; 1.1
	3	1.1	3.3; 2.2
Number of SR-links (J)	1	361	1083
	2	361	722
	3	361	722
Time gates t (ps)	1	200	200, 133, 66
	2	200	200, 100
	3	200	100, 66
Photon average velocity v (mm/ps)	1	0.0165	0.0165
	2	0.011	0.011
	3	0.0055	0.033
True value of FPDF f (${\mathrm{mm}}^{-1}$)	1	0.001	0.001
	2	0.0014	0.0014
	3	0.0018	$4.2\cdot 10^{-4}$

### 3.4 Results and their Analysis

In this section, results of FPDF reconstruction with strategies 1 and 2, and results of fluorescence parameter distribution separation by the method described in Sec. 2.4 are presented.

**Fig. 4:**
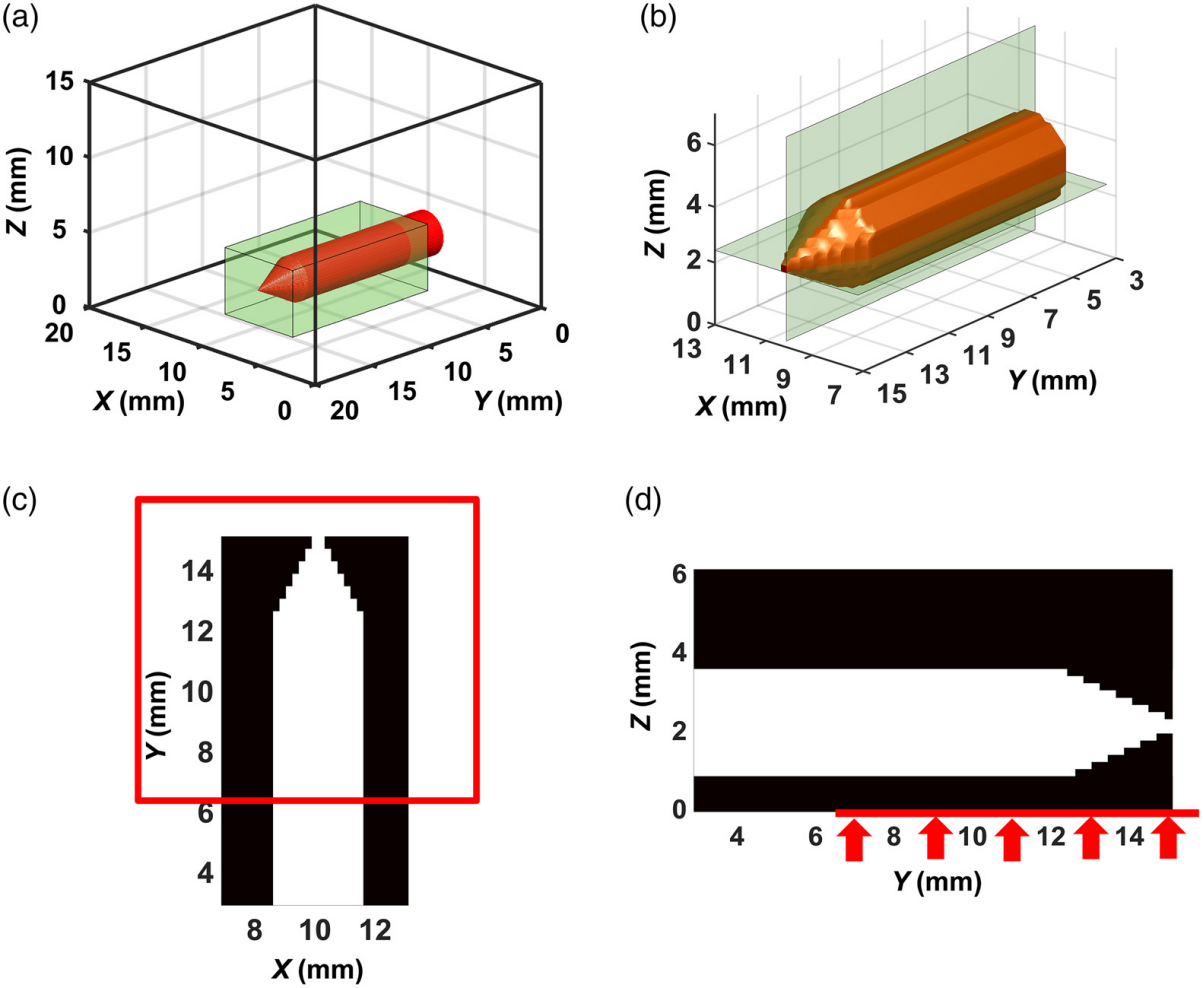
Numerical model of the phantom with the fluorophore: (a) the entire ROI to be reconstructed; (b) its enlarged part to be visualized; (c) Z-section of the part; (d) X-section.

Figure 4 shows a view of the numerical model of the phantom, which represents one of the FPDF distributions. The entire ROI we were reconstructing is shown in Fig. 4(a). The green rectangle in Fig. 4(a) defines the “useful” part of ROI with the fluorophore, which is directly adjacent to the scan area of $9\times 9\;{\mathrm{mm}}^{2}$ and below visualized in the figures with reconstruction results. Figure 4(b) presents this part in an enlarged format and identifies the horizontal (Z-section) and vertical (X-section) planes, which are below used to visualize reconstruction results in 2D sections of the “useful” part [Figs. 4(c) and 4(d)]. Figures 4(c) and 4(d) also show in red the boundaries of the scan area in the XOY plane.

**Fig. 5:**
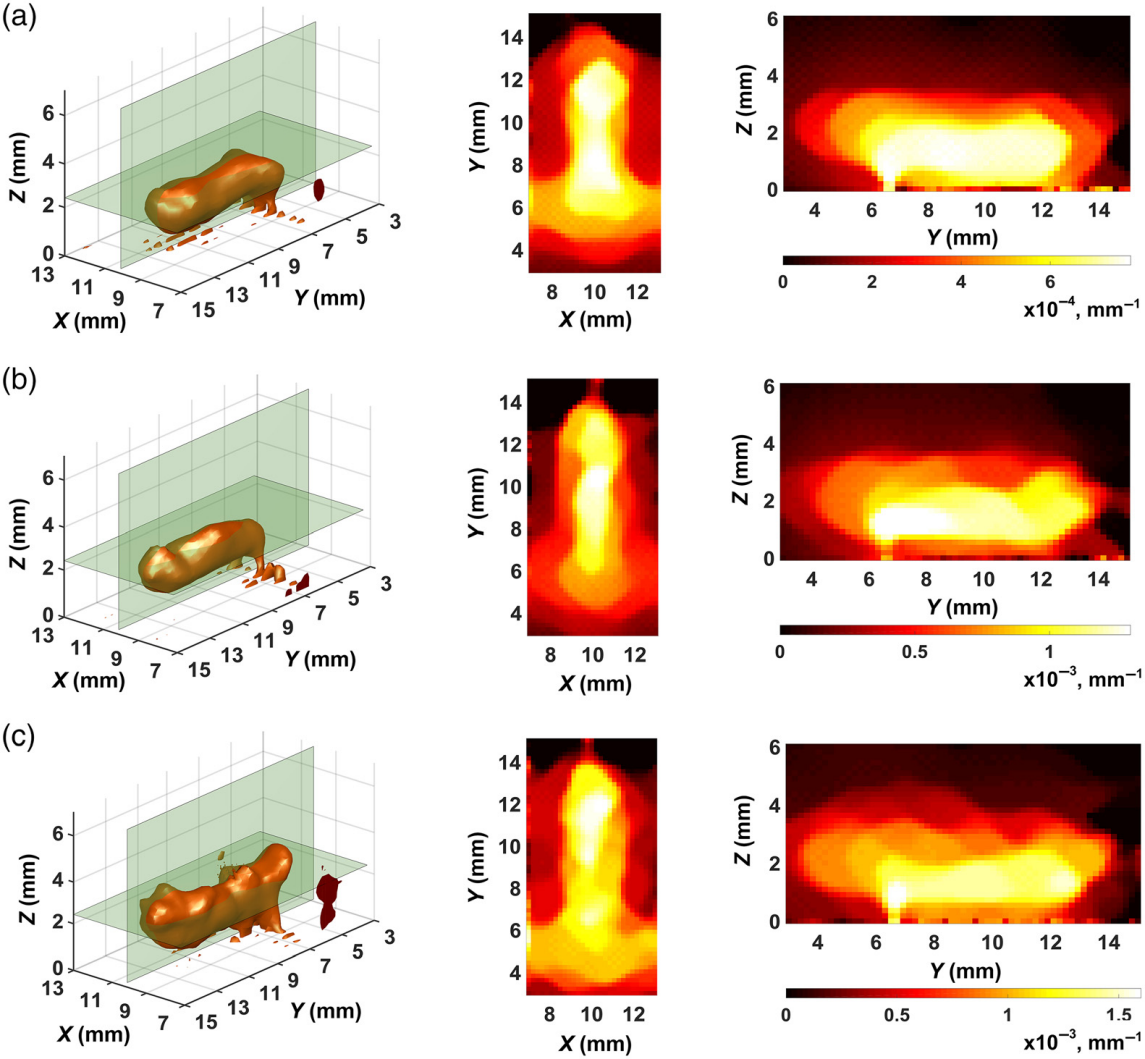
FPDF reconstructions with the use of strategy 1: (a) reconstruction No. 1; (b) reconstruction No. 2; (c) reconstruction No. 3.

**Fig. 6:**
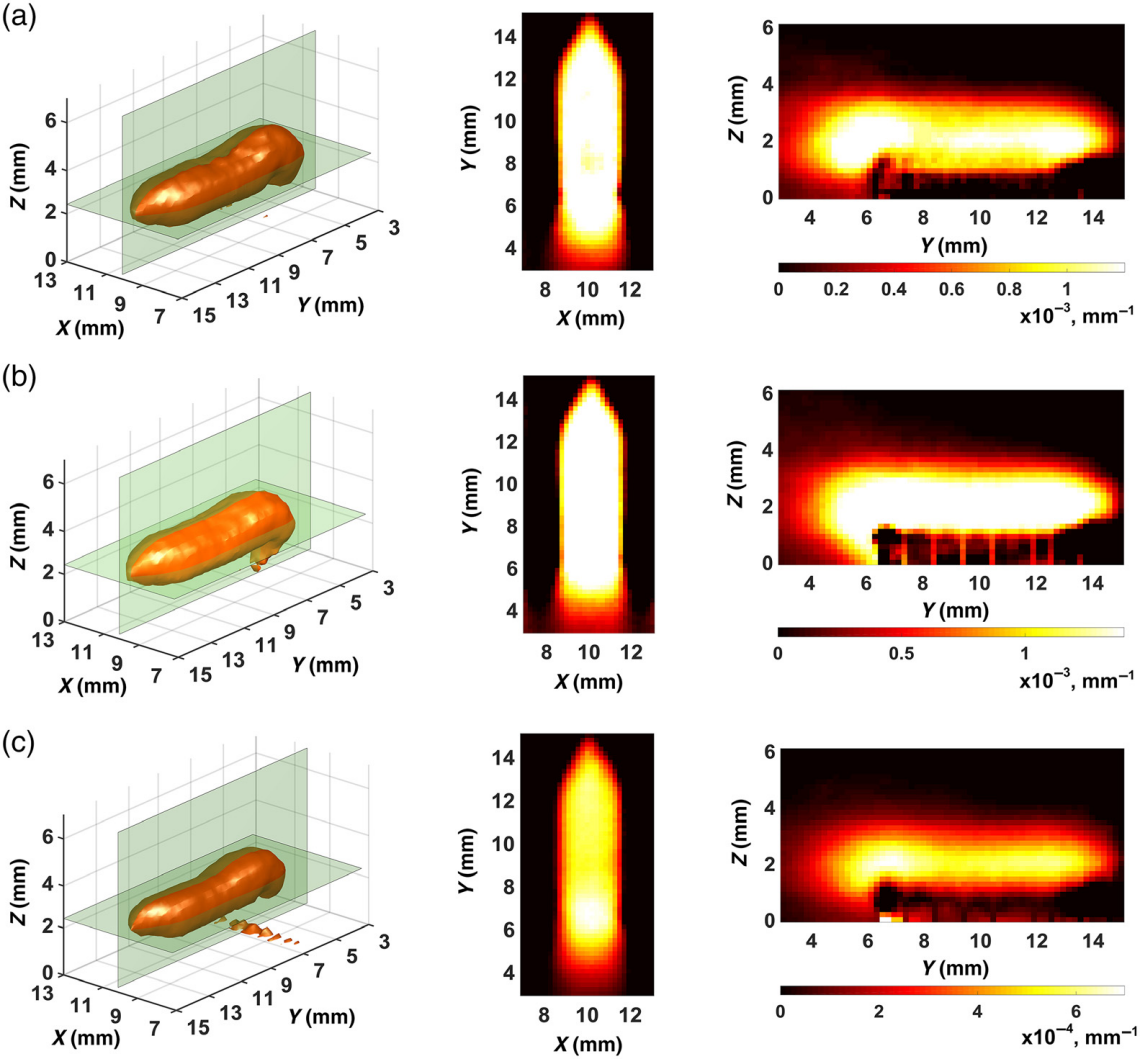
FPDF reconstructions with the use of strategy 2: (a) reconstruction No. 1; (b) reconstruction No. 2; (c) reconstruction No. 3.

Figures 5 and 6 show the best results we obtained in the reconstruction of FPDFs with strategies 1 and 2, respectively. In all cases, the ART-FIST-TV algorithm described in Sec. 2.3 was used for reconstruction. As noted above (see Fig. 4 and its description), the phantom tomogram parts with useful information (“useful” parts) are only visualized in the figures. In each of Figs. 5(a)–5(c) and Figs. 6(a)–6(c), a 3D view of the part is shown on the left, and the other two views show its 2D Z- and X-sections in the center and on the right, respectively. The palette scales are graduated in inverse millimeters.

Visual analysis of the tomograms presented in Figs. 5 and 6 shows that the images in Fig. 6 look much clearer, contain fewer artifacts (mostly outside the scan area) and better reproduce the true FPDF values than the images in Fig. 5. Thus, the visual comparison of the FPDF reconstructions allows us to suppose the superiority of strategy 2 (Fig. 6) over strategy 1 (Fig. 5). It should, however, be noted that such a comparison at the stage of FPDF reconstruction is not critical since the main goal is to separate fluorescence parameters. That is the reason why we analyzed the tomograms of Figs. 5 and 6 only visually with no use of quantitative characteristics.

**Fig. 7:**
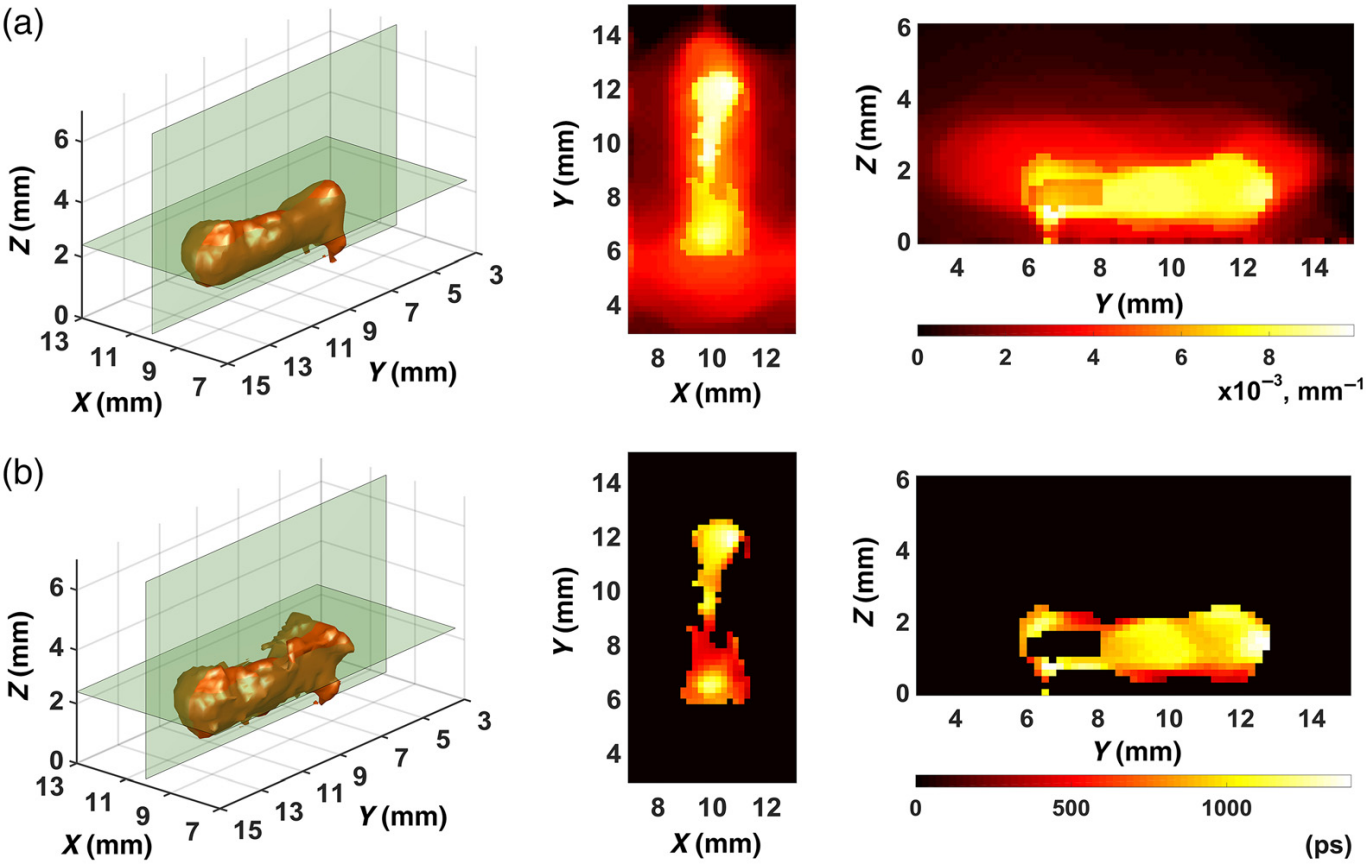
Fluorescence parameter separation results for strategy 1: (a) fluorophore absorption coefficient $\mu_{af}(r)$; (b) fluorescence lifetime τ(r).

**Fig. 8:**
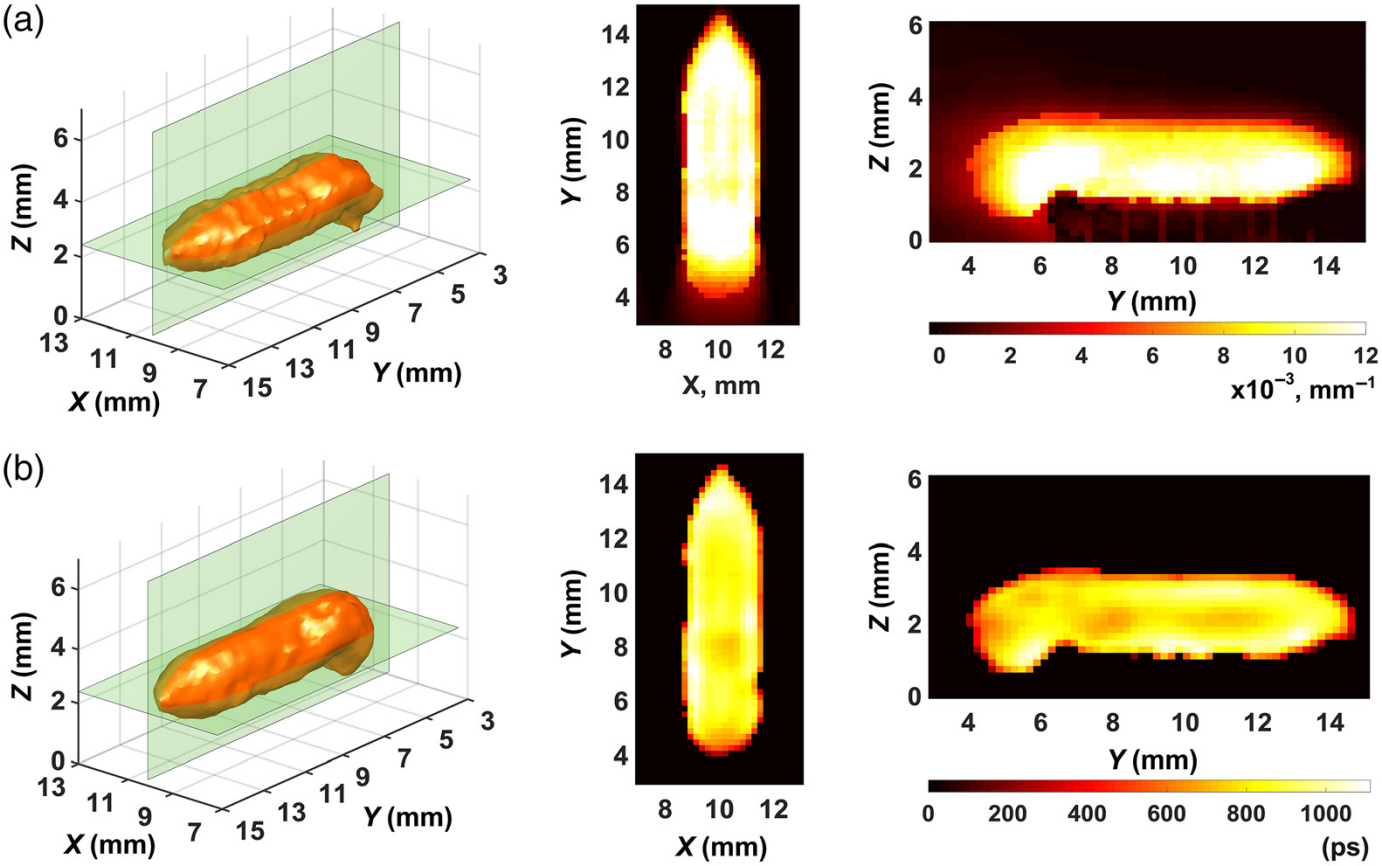
Fluorescence parameter separation results for strategy 2: (a) fluorophore absorption coefficient $\mu_{af}(r)$; (b) fluorescence lifetime τ(r).

Figures 7 and 8 present what we obtained in the separation of the fluorophore absorption coefficient $\mu_{\mathrm{af}}(r)$ and fluorescence lifetime τ(r) distributions with strategies 1 and 2, respectively. The method of Sec. 2.4 was used. The distributions $\mu_{af}(r)$ and τ(r) were visualized in the same manner as the FPDF distributions (Figs. 5 and 6). The palette scale is in inverse millimeters for the absorption coefficient [Figs. 7(a) and 8(a)] and in picoseconds for lifetime [Figs. 7(b) and 8(b)]. The visual comparison of Figs. 7 and 8 shows that both the fluorophore absorption coefficient and the fluorescence lifetime are reconstructed much better with strategy 2 (Fig. 8). The images of Fig. 7 look blurred and very incorrectly reproduce the shape of the fluorophore. Image intensities in Fig. 7(b) differ to such an extent that it is impossible to see that the nominal value of the lifetime is equal to 900 ps. So, the result obtained with strategy 1 must be deemed unsatisfactory, whereas with strategy 2 the reconstructed lifetime distribution [Fig. 8(b)] has rather sharp-cut boundaries and correctly, on average, reproduces the nominal lifetime value. Thus, the visual analysis of results we obtained in fluorescence parameter separation allows us to say that strategy 2 outperforms strategy 1.

To prove this conclusion quantitatively, we calculated such characteristics of image quality as the correlation coefficient $k_{\mathrm{cor}}$ and the deviation factor $k_{\mathrm{dev}}$ for the images of Figs. 7 and 8 by the equations

$$k_{\mathrm{cor}}=\frac{\overset{I}{\underset{i=1}{\sum }}(x_{i}^{\mathrm{tom}}-{\overset{¯}{x}}^{\mathrm{tom}})(x_{i}^{\mathrm{src}}-{\overset{¯}{x}}^{\mathrm{src}})}{(I-1)\Delta x^{\mathrm{tom}}\Delta x^{\mathrm{src}}},$$ (25)

and

$$k_{\mathrm{dev}}=\frac{\sqrt{\frac{1}{I}\overset{I}{\underset{i=1}{\sum }}{\left(x_{i}^{\mathrm{tom}}-x_{i}^{\mathrm{src}}\right)}^{2}}}{\Delta x^{\mathrm{src}}},$$ (26)

where $\overset{¯}{x}$ and Δx are the mean and the root-mean-square deviation over all I voxels of image x, and the indices “src” and “tom” relate them to the phantom model and the reconstructed image, respectively. Our experience (see, e.g., Refs. 30 and 59) tells of convenience and efficiency to use these characteristics in cases where *a priori* information about the object is known and its exact model can be derived. If $k_{\mathrm{cor}}$ is close to unity, the model and its reconstruction are highly correlated pointing to high reconstruction accuracy. If $k_{\mathrm{dev}}$ is close to zero, the two images agree well and reconstruction accuracy is also high. If is closer to zero than to unity and is close to or greater than unity, then reconstruction quality is bad. The calculated values of and are presented in Table 2. In Table 2, the abnormal values of the correlation coefficient and deviation factor obtained for the fluorescence lifetime distribution with strategy 1 are highlighted in bold.

**Table 2:** Quantitative characteristics for the images of Figs. 7 and 8.

Quantitative characteristics	Strategy No.	Figure No.	Value
Correlation coefficient $k_{\mathrm{cor}}$	1	7A	0.6355
		7B	**0.3804**
	2	8A	0.7946
		8B	0.7419
Deviation factor $k_{\mathrm{dev}}$	1	7A	0.7886
		7B	**0.9612**
	2	8A	0.5462
		8B	0.6751

So, the data of Table 2 confirm the above conclusion that strategy 2 works better than strategy 1. This means that using the results of phantom scanning data processing, we succeeded to prove the above hypothesis that as many SR-links as possible should be used for FPDF reconstruction.

All calculations related to experimental data processing (except for simulation of sensitivity functions and FTPSFs) were done on an Intel PC with a 3.4 GHz i5 processor and 8 GB RAM in MATLAB. Among all operations, FPDF reconstruction was most time demanding: about 1 h for the sensitivity matrix of size 361×6000000 and about 2.5 hours for the matrix of size 1083×6000000.

## 4 Discussion

The proposed new mesoscopic FMLT method with the use of early arriving photons was tested against experimental data obtained by scanning a fluorescent phantom with a three-channel fiber-optic probe. At this stage, only one scheme was used for carrying out spatially dependent time-resolved measurements, which can hardly be considered optimal from a geometric point of view. An absolutely positive fact is that even for as small as only three different SR distances, it appeared possible to find such a strategy for the formation of the measurement data arrays and sensitivity matrices that ultimately helped to adequately reconstruct the distribution of the fluorescence lifetime. However, to achieve the result, *a priori* information about the parameters of the phantom was used, and this was done on purpose. The main goal was to measure the potential of the proposed approximated method based on the use of only early arriving fluorescent photons and find its limit “from above.” With respect to *a priori* information, first, sensitivity functions were modeled and then sensitivity matrices were formed with allowance for the found dependence of the functions on the relative position of fluorophore and source–receiver pairs. Second, *a priori* knowledge was used for modeling FTPSFs in order to measure the true time shift of processed fluorescent responses from the time origin. It is clear that in the study of tumors in laboratory animals, *a priori* information on geometrical, optical, and fluorescence parameters will be rather limited. To measure the potential of the proposed method but now “from below,” we found it appropriate to perform two more series of FPDF reconstructions with further separation of fluorescence parameter distributions. In both cases measurement data and sensitivity matrices were formed with strategy 2.

**Fig. 9:**
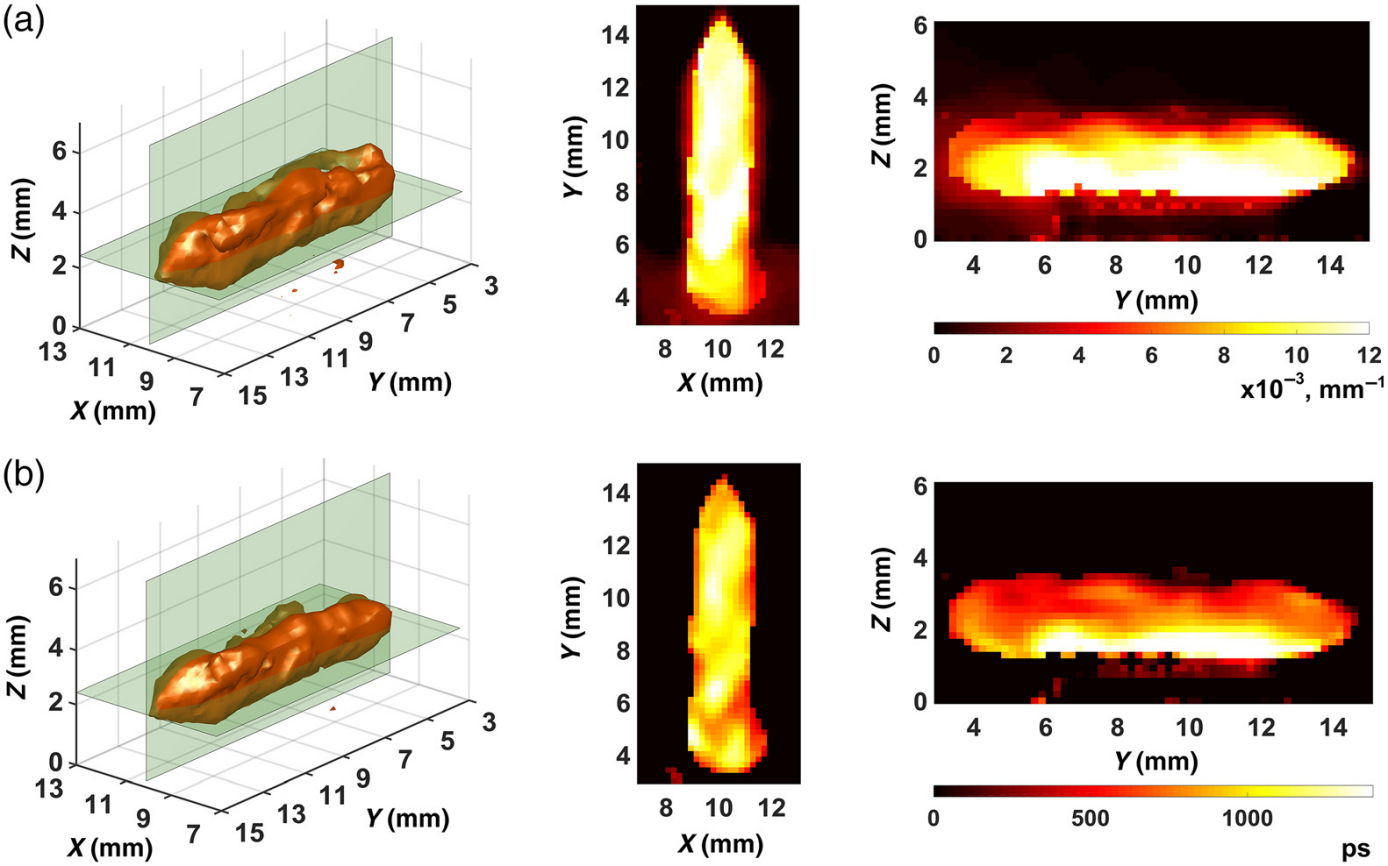
Results of fluorescence parameter separation for “symmetric” sensitivity functions: (a) fluorophore absorption coefficient; (b) fluorescence lifetime.

In the first case, “symmetric” functions were used instead of the space-dependent sensitivity functions. One of them is shown in Fig. 1(c). To claim adequate results, we crudely calibrated these functions in amplitude through multiplication by a multiple factor based on available experimental evidence. That is, in the sensitivity matrices, for each SR-link characterized by the same SR-distance, “symmetric” sensitivity functions were written with multiple intensity values. The measurement data arrays were however formed with account for *a priori* information, i.e., the true time shifts of FTPSFs determined through modeling. The final result of $\mu_{\mathrm{af}}(r)$ and τ(r) separation is shown in Fig. 9. Visualization is done in the same manner as in Figs. 7 and 8.

**Fig. 10:**
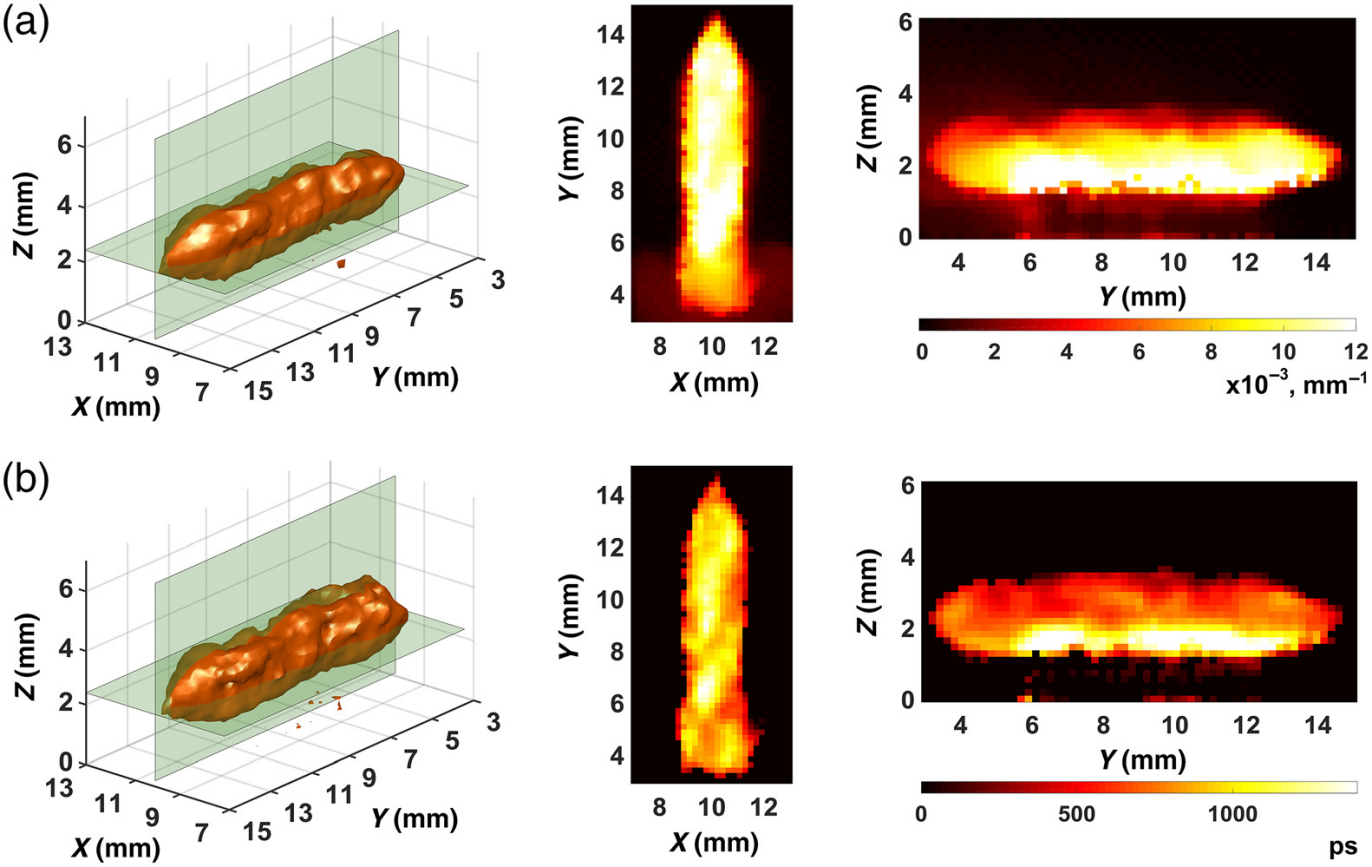
Results of fluorescence parameter separation with no use of *a priori* information: (a) fluorophore absorption coefficient; (b) fluorescence lifetime.

In the second series of reconstructions, the sensitivity matrices were also based on the “symmetric” sensitivity functions. Furthermore, data from FTPSF simulations by TurbidMC were not used for forming measurement data arrays. The time origin was not associated with the beginning of the excitation pulse, as we did earlier, but was defined conventionally by the method described in Ref. 12 on the basis of IRF measurements. As a result, each of the measured or processed FTR had its own binding to IRF and the experimentally evaluated time origin. Our analysis of model FTPSFs showed that errors in the counting of the time gates chosen in strategy 2 were within 0 to 15 ps, which corresponded to measurement data errors within 0% to 8%. Thus the second series of calculations corresponded to a situation where *a priori* information about the object was not used at all. The final result of $\mu_{\mathrm{af}}(r)$ and τ(r) separation for this case is presented in Fig. 10. Table 3 provides $k_{\mathrm{cor}}$ and $k_{\mathrm{dev}}$ for the images of Figs. 9 and 10.

**Table 3:** Quantitative characteristics for the images of Figs. 9 and 10.

Quantitative characteristics	Prior for generation of W and g	Figure No	Value
Correlation coefficient	W – no	9A	0.7275
	g – yes	9B	0.6797
	W – no	10A	0.7012
	g – no	10B	0.6465
Deviation factor	W – no	9A	0.6574
	g – yes	9B	0.7379
	W – no	10A	0.6857
	g – no	10B	0.7613

Comparative analysis of data presented in Figs. 8–10 and Tables 2 and 3 shows that, as expected, the quality of tomograms presented in Figs. 9 and 10 is lower than that of images in Fig. 8. This is to a greater extent true for the tomograms of Fig. 10 and to a smaller extent to those of Fig. 9. So, in both Figs. 9 and 10, the fluorophore boundaries look more blurred and the nominal lifetime (900 ps) is reproduced with the greater error than in Fig. 8. At the same time the reconstructions presented in Figs. 9 and 10 are clearly better than the reconstruction of Fig. 7 where strategy 1 was used.

This means that the proposed mesoscopic FMLT method based on early arriving photons is capable of giving adequate results even without *a priori* information about the object due to the properly chosen strategy for forming the arrays g and W. It is however very desirable to use this information because it helps attain better quality of reconstruction.

What initial information can be available in the study of tumors implanted in small animals? And what are the prospects of its effective use with the proposed method? First, it seems quite reasonable to assume that the tumor is implanted at a known site underneath the skin. Its approximate shape (say, almost spherical) and size (say, about 4 to 5 mm) can also be known. If this information is absent, it can be recovered through multimodal imaging, e.g., by adding such a modality as magnetic resonance imaging (MRI). In recent decades, the hybrid FMT/MRI systems have increasingly been applied for small animal imaging.^67^^–^^69^ Just MRI is one of high-resolution modalities that provide useful structural information about tumor localization. Moreover, when, in particular, the multimodal contrast agents are applied,^70^ not only structural information but also functional and molecular one can be obtained with MRI. That is why the hybrid FMLT/MRI approach is marled for a great future. The presence of initial structural information means that the ROI to be reconstructed with the proposed method can be chosen conveniently, e.g., so that the tumor occupies the most of ROI volume and is located in its central part. In this case, we can assume that the dependence of sensitivity functions on fluorophore localization will be fully devalued and will not influence the result of reconstruction.

Second, it is quite possible to determine the initial range of values for the optical and fluorescence parameters of the tumor and even define their initial approximations $D^{\left(0\right)},c^{\left(0\right)},\gamma^{\left(0\right)},\mu_{af}^{\left(0\right)},\tau^{\left(0\right)}$. Fluorescence parameters, as well as the shape and size of the fluorophore region, can be adjusted through a multistep approach similar to that proposed in Refs. 21 and 22. Adjustment algorithms may differ. Below described is a very simple algorithm that consists of two steps.

The first step involves adjustment of the fluorophore absorption coefficient $\mu_{af}^{\left(1\right)}(r)$ with use of a reconstruction model, which is described by the equation

$$\Gamma^{f}(r_{s},r_{d},t)∼\int_{V}[\frac{4D^{\left(0\right)}c^{\left(0\right)}\gamma^{\left(0\right)}}{\tau^{\left(0\right)}v^{2}(t)+4D^{\left(0\right)}c^{\left(0\right)}}\cdot W_{f}^{\left(0\right)}(r_{s},r_{d},r,t)]\mu_{af}^{\left(1\right)}(r)d^{3}r,$$ (27)

where the expression in the square brackets is the sensitivity function responsible for the reconstruction of $\mu_{af}^{\left(1\right)}(r)$, and $W_{f}^{\left(0\right)}(r_{s},r_{d},r,t)$ is modeled in accordance with Eq. (9) for the chosen initial values of parameters. At this step, the shape and size of the fluorophore region $V_{\mathrm{flu}}$ are adjusted and the mean value $\mu_{\mathrm{af}}^{\left(1\right)}=\langle \mu_{\mathrm{af}}^{\left(1\right)}(r)\rangle_{r\in V_{\mathrm{flu}}}$ is determined. At the second step we adjust the lifetime $\tau^{\left(1\right)}(r)$, e.g., by inverting the equation

$$\Gamma^{f}(r_{s},r_{d},t)∼\int_{V}W_{f}^{\left(1\right)}(r_{s},r_{d},r,t)f^{\left(1\right)}(r)d^{3}r,$$ (28)

for the function

$$f^{\left(1\right)}(r)=\frac{4D^{\left(0\right)}c^{\left(0\right)}\gamma^{\left(0\right)}\mu_{af}^{\left(1\right)}}{\tau^{\left(1\right)}(r)v^{2}(t)+4D^{\left(0\right)}c^{\left(0\right)}},$$ (29)

where $W_{f}^{\left(1\right)}(r_{s},r_{d},r,t)$ is sensitivity function Eq. (9) simulated for the parameters adjusted at the first step. The adjusted data can be used to calculate the sensitivity functions and FTPSFs, which will then be used to reconstruct FPDFs and to separate the distributions of fluorescence parameters. So, from the above reasoning we can guess that the information on the object, which is needed for the effective application of the proposed method can be collected *a posteriori* after a number of tentative (adjusting) reconstructions. Verification of this hypothesis in experiments with animals is the subject of our research in the near future.

## 5 Conclusion and Future Research

A method of mesoscopic time-domain fluorescence molecular tomography has been presented. It uses only the early arriving diffuse photons and helps to separate the spatial distributions of the fluorophore absorption coefficient and fluorescence lifetime directly in the time domain. The method is based on the asymptotic approximation of the fluorescence source function, which makes it possible to determine the fluorescence parameter distribution function in a simple form comprising both the absorption coefficient distribution and the fluorescence lifetime distribution. The inverse tomography problem is solved exactly for this function and then the distributions are separated by solving an overdetermined system of algebraic equations.

We tested the method against experimental data from scanning a phantom with a fluorophore by a three-channel fiber probe in reflectance geometry. The fluorescence parameter distribution function was reconstructed with a hybrid compressed-sensing-like algorithm ART-FIST-TV, which combines algebraic reconstruction, fast iterative shrinkage thresholding, and total variation regularization. The distributions were separated with the known QR-factorization least square algorithm. It is shown that in case where *a priori* information on the object is available, fluorescence parameters can be reconstructed quite adequately by choosing a proper strategy for the generation of early-time-gate-based measurement data arrays and sensitivity matrices. The proposed method however appeared sensitive to errors in the definition of the time origin for time gate counts for forming measurement data arrays. That is why in case where *a priori* information on the object is limited, it seems appropriate to do tentative reconstructions in order to refine object parameters and collect additional *a posteriori* information, which can effectively be used in the method. Development and testing of such gauge methods and algorithms, in particular, through experiments with animals is the subject of our research in the near future.

It can be noted that the question of the spatial resolution of the proposed method remains open. Unfortunately, it cannot be estimated using the investigated phantom. Such an assessment requires special phantoms with small fluorescent inclusions that form periodic spatial structures,^10^^,^^16^^,^^17^^,^^19^^–^^21^^,^^29^^,^^30^ or phantoms that simulate the volumetric structure of the vascular bed.^52^^,^^54^^,^^71^ However, it should be understood that we are in principle incapable of getting high-resolution images with a fiber 0.4 mm in diameter. Not only the phantom needs modernization but also equipment and data registration geometry. Recent studies in mesoscopic fluorescence molecular tomography^52^^,^^54^^,^^71^ and high-density diffuse optical tomography^72^^–^^74^ suggest that the quality of diffuse tomograms can be improved if increase the density of sources and receivers, extend the range of source–receiver distances, and use different “crossing” source–receiver links. So, we think it appropriate to change to noncontact illumination and data registration, TCSPC equipment with better time resolution, geometry of type “high-density diffuse optical tomography”, and a fine-dispersed phantom to test the spatial resolution. Also, in our view the combination of meso- and macro-regimes as well as reflection and transmission geometries is more promising. Such an approach is likely to help escape form the pronounced spatial variance of images. All this is included in our short-term plans.

## 6 Appendix: Reconstruction Algorithm

Let λ be the control parameter of ART iterations, α be the regularization parameter, be the iteration number of the ART-FIST cycle, β be the step of the gradient descent iteration, $S_{\mathrm{tv}}$ be the iteration number of the TV cycle, ${\mathrm{ART}}_{\lambda }(\cdot )$ be the operator that performs the cycle of standard ART iterations with single search over all SR-links, and ${\text{Shrink}}_{\alpha ,\lambda }(\cdot )$ be the operator that performs image shrinkage in accordance with the algorithm from Ref. 56. Then the ART-FIST-TV algorithm can be described by the following successive steps:

**ART-FIST-TV algorithm**

Step 1. Initialize initial approximation $f^{\left(0\right)}$, ART-FIST cycle parameters λ, α, $S_{\mathrm{art}-\mathrm{fist}}$ and TV cycle parameters β.
Step 2. Set $y^{\left(1\right)}=f^{\left(0\right)}$, $t^{\left(1\right)}=1$.
Step 3. Do iterations of the ART-FIST cycle by the formulas:
  $$f^{\left(s\right)}={\text{Shrink}}_{\alpha ,\lambda }[{\mathrm{ART}}_{\lambda }(y^{\left(s\right)})],$$
  $$t^{\left(s+1\right)}=\frac{1+\sqrt{1+4{\left[t^{\left(s\right)}\right]}^{2}}}{2},$$
  $$y^{\left(s+1\right)}=f^{\left(s\right)}+\frac{t^{\left(s\right)}-1}{t^{\left(s+1\right)}}[f^{\left(s\right)}-f^{\left(s-1\right)}].$$
Step 4. Set $f^{\left(0\right)}=y^{\left(S_{\mathrm{art}-\mathrm{fist}}\right)}$.
Step 5. Do $S_{\mathrm{tv}}$ iterations of the TV cycle in by the equation
  $$f_{i}^{\left(s+1\right)}=f_{i}^{\left(s\right)}-\beta \frac{\partial {\left\|f^{\left(s\right)}\right\|}_{\mathrm{TV}}}{\partial f_{i}},$$
  where ${\left\|\cdot \right\|}_{\mathrm{TV}}$ is the total variation norm.
Step 6. Check the stop criterion. If not satisfied, set $f^{\left(0\right)}=f^{\left(S_{\mathrm{tv}}\right)}$, $y^{\left(1\right)}=f^{\left(S_{\mathrm{tv}}\right)}$, $t^{\left(1\right)}=1$ and go to step 3.
Step 7. End calculations if the stop criterion is satisfied.

As for the stop criterion, we finish the iterative process when there are no appreciable changes in the images from the previous and current iterations of the external cycle (ART-FIST plus TV). Initial approximation $f^{\left(0\right)}$ and parameters of ART-FIST and TV cycles were taken to be the same as in Ref. 30 and had the following values: $f^{\left(0\right)}=0$, λ=0.9, α=0.001, $S_{\mathrm{art}-\mathrm{fist}}=50-150$, $S_{\mathrm{tv}}=3-5$. As for the step of the gradient descent iteration β, it had initially a maximal value of 0.005 and then adjusted each iteration through multiplication by 0.997.

## Code, Data, and Materials Availability

The code and data that support the findings of this study are available from the corresponding author upon reasonable request.
